# Exploring Cognitive and Affective Outcomes Following Bilateral 6‐Hydroxydopamine Lesions of the Substance Nigra or Striatum in Rats

**DOI:** 10.1002/jnr.70154

**Published:** 2026-09-07

**Authors:** Bianca Andretto de Mattos, Elaine Del Bel, Humberto Milani, Rúbia Maria Weffort de Oliveira, Jéssica Mendes Bonato

**Affiliations:** ^1^ Department of Pharmacology and Therapeutics State University of Maringá Maringá Paraná Brazil; ^2^ Department of Physiology School of Medicine of Ribeirão Preto Ribeirão Preto São Paulo Brazil; ^3^ Department of Basic and Oral Biology School of Odontology of Ribeirão Preto Ribeirão Preto São Paulo Brazil

**Keywords:** animals, behavior, hippocampal neurogenesis, nonmotor symptoms, ventral tegmental area

## Abstract

The majority of animal models of Parkinson's disease (PD) focus on motor symptoms that are induced by unilateral injections of such neurotoxins as 6‐hydroxydopamine (6‐OHDA) in nigrostriatal dopaminergic pathways. However, motor changes that are induced by unilateral 6‐OHDA injections may interfere with the identification of cognitive and affective dysfunctions induced by dopaminergic neurodegeneration. To select an appropriate method for studying nonmotor symptoms of PD and potential neuroprotective treatments, the present study compared behavioral effects of bilateral 6‐OHDA infusions directly in the substantia nigra pars compacta (SNpc) or striatum in rats. A battery of behavioral tests, including affective and cognitive tasks, was performed for 22 days after 6‐OHDA nigrostriatal lesions. The massive degeneration of tyrosine hydroxylase‐immunoreactive neurons was observed in the SNpc, striatum, and ventral tegmental area with 6‐OHDA infusions in either the SNpc or striatum. Concerning functional outcomes, 6‐OHDA infusions in the striatum decreased general exploratory activity 7 days after the lesion. Rats that received 6‐OHDA in the SNpc exhibited cognitive impairments and despair‐like behavior. A decrease in the number of newborn neurons was found in the hippocampus in rats that received 6‐OHDA in the striatum, indicating a alteration of neuron maturation. 6‐OHDA infusions in both the SNpc and striatum impacted the maturation of newborn hippocampal neurons. These results indicate that bilateral injections of 6‐OHDA in the SNpc might be useful for studying nonmotor symptoms of PD.

## Significance Statement

Finding a study that compares different methods to determine the most appropriate choice for the research objective is not always easy. Therefore, comparing methods and their results is highly significant. To select an appropriate method for studying the non‐motor symptoms of PD and potential neuroprotective treatments, the present study compared the behavioral effects of bilateral 6‐OHDA infusions into the SNpc or striatum of rats, as well as the loss of dopaminergic neurons in the SNpc, ventral tegmental area (VTA), and striatum, to demonstrate the essential differences between lesions.

## Graphical abstract

Our results demonstrate massive degeneration of tyrosine hydroxylase‐immunoreactive neurons was observed in the SNpc, striatum, and ventral tegmental area with 6‐OHDA infusions in either the SNpc or striatum. Rats administered 6‐OHDA to the SNpc exhibited cognitive impairments, anxiety‐like behaviors, and impacted the maturation of newborn hippocampal neurons.

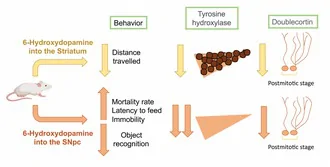

## 1 Introduction

Parkinson's disease (PD) is a chronic neurodegenerative condition that affects 1% of the population above 60 years of age (Tysnes and Storstein 2017), and more than 10 million people are predicted to be affected worldwide by 2030 (Blesa et al. 2022). PD is mainly characterized by the progressive loss of dopaminergic projection neurons from the substantia nigra pars compacta (SNpc) to the striatum. Classically, PD includes such motor symptoms as bradykinesia, resting tremors, rigidity, and postural disturbances (Schapira et al. 2017; Blesa et al. 2022). Patients with PD also exhibit disabling nonmotor symptoms. In many cases of PD, these symptoms are scarcely recognized and reported and thus insufficiently treated (Poewe et al. 2017; Patricio et al. 2022). The main nonmotor symptoms of PD severely affect PD patients' quality of life and include sleep disorders, olfactory and autonomic dysfunction, fatigue, pain, cognitive deficits, anxiety, and depression (Kalia and Lang 2016). Anxiety is present in approximately 40%–60% of PD patients and appears to be commonly associated with depression (Martínez‐Martín and Damián 2010; Schapira et al. 2017). Depression occurs in approximately 35% of PD patients and most often involves such symptoms as apathy and anhedonia (Aarsland et al. 2011). Cognitive impairment is seen as a progressive sign of PD and is present in 25%–30% of patients (Kehagia et al. 2010; Schapira et al. 2017; Hayes et al. 2019).

The causes of nonmotor symptoms of PD are multifactorial and poorly known. Patients who do not respond to classic dopaminergic anti‐PD drugs experience difficult and insufficient management of their symptoms (Poewe et al. 2017; Santos García et al. 2019). These nonmotor symptoms appear to be related to dopaminergic, serotonergic, and noradrenergic neurotransmission (Santiago et al. 2010), and a decline of hippocampal synaptic plasticity (Marxreiter et al. 2013; Winner and Winkler 2015; Györfi et al. 2017; Lim et al. 2018). Notably, dementia (Camicioli et al. 2003), cognitive impairments (Ermine et al. 2018), and depressive symptoms have been linked to a reduction of neurogenesis in patients with PD. In rats, 6‐hydroxydopamine (6‐OHDA)‐induced nigral lesions reduced cell proliferation in the subgranular zone (SGZ) of the hippocampus (Suzuki et al. 2010). This is particularly important considering the roles of the hippocampus in cognition and mood regulation.

Administration of the toxin 6‐OHDA in brain regions in rodents, including the SNpc, medial forebrain bundle (MFB), and striatum, has been widely reported to cause the degeneration of dopaminergic neurons and mimic some clinical aspects of PD (Simola et al. 2007; Chia et al. 2020). The magnitude of the lesion and consequent symptoms depend on the dose of 6‐OHDA that is injected, the site of the lesion, uni‐ and bilateral lesions, and the age and species of animals (Betarbet et al. 2000; Tieu 2011). When injected in the MFB, 6‐OHDA produces a rapid and complete lesion in the nigrostriatal pathway, with massive dopaminergic neuron degeneration within 12 h. This degeneration precedes the loss of striatal terminals, which occurs 2–3 days later (Jeon et al. 1995; Sarre et al. 2004; Blandini and Armentero 2012). 6‐OHDA microinjections in the striatum induce slow, progressive, and partial retrograde damage to the nigrostriatal pathway for up 3 weeks (Sauer and Oertel 1994; Przedbroski et al. 1995). Intrastriatal lesions are consistent with the axonal terminal degeneration “dying back” theory of PD (Burke and O'Malley 2013). However, few studies have used bilateral injections of 6‐OHDA in the SNpc or striatum to evaluate nonmotor symptoms of PD, despite the fact that bilateral lesion models mimic the human condition (Deumens et al. 2002). Moreover, the results of available studies have been conflicting (Branchi et al. 2008; Tadaiesky et al. 2022).

To select an appropriate method for studying nonmotor symptoms of PD and potential neuroprotective treatments, the present study compared behavioral effects of bilateral 6‐OHDA infusions in either the SNpc or striatum in rats. Dopaminergic neuron loss was quantified by determining tyrosine hydroxylase (TH) immunoreactivity in the SNpc, ventral tegmental area (VTA), and striatum. Behavioral dysfunction was estimated using a battery of tests, including the open field test (OFT), object recognition test (ORT), splash test (ST), novelty‐suppressed feeding test (NSF), and forced swim test (FST). To evaluate dopaminergic lesions that influence hippocampal DCX cell population, the expression of newborn hippocampal neurons was evaluated by the immunohistochemical detection of doublecortin (DCX)‐immunoreactive (IR) neurons.

## 2 Materials and Methods

### 2.1 Animals

Male 3‐month‐old Wistar rats (280–320 g) were obtained from the central vivarium of the State University of Maringá. The animals were acclimated to a controlled temperature (22°C ± 1°C) and a 12 h/12 h light/dark cycle (lights on at 7:00 AM) for 2 weeks before the experiments. The animals were housed in groups (*n* = 5–10/group) in plastic cages and given standard commercial chow (Nuvilab, Quimtia, PR, Brazil) and tap water *ad libitum*. The local Ethics Committee on Animal Experimentation of the State University of Maringá approved the experimental procedures according to the guidelines of the US National Institutes of Health and Brazilian College for Animal Experimentation (CEUA no. 8156290119). All efforts were made to minimize the number of animals used and their suffering.

### 2.2 Drugs

Desipramine and 6‐OHDA were obtained from Sigma‐Aldrich (Sigma, St. Louis, MO, USA). Desipramine was dissolved in sterile saline, and 6‐OHDA was dissolved in vehicle (sterile saline with 0.2% ascorbic acid). 6‐OHDA was infused bilaterally in a single dose directly in the SNpc (4 μg/μl) or dorsolateral striatum (12 μg/μl). 6‐OHDA was prepared according to established doses that were shown to promote significant dopaminergic neuron loss in the SNpc (Santiago et al. 2014; Bonato et al. 2018, and de Mattos et al. 2024) and striatum (Rampersaud et al. 2012; Shobana et al. 2012).

### 2.3 Stereotaxic Surgery

**FIGURE 1:**
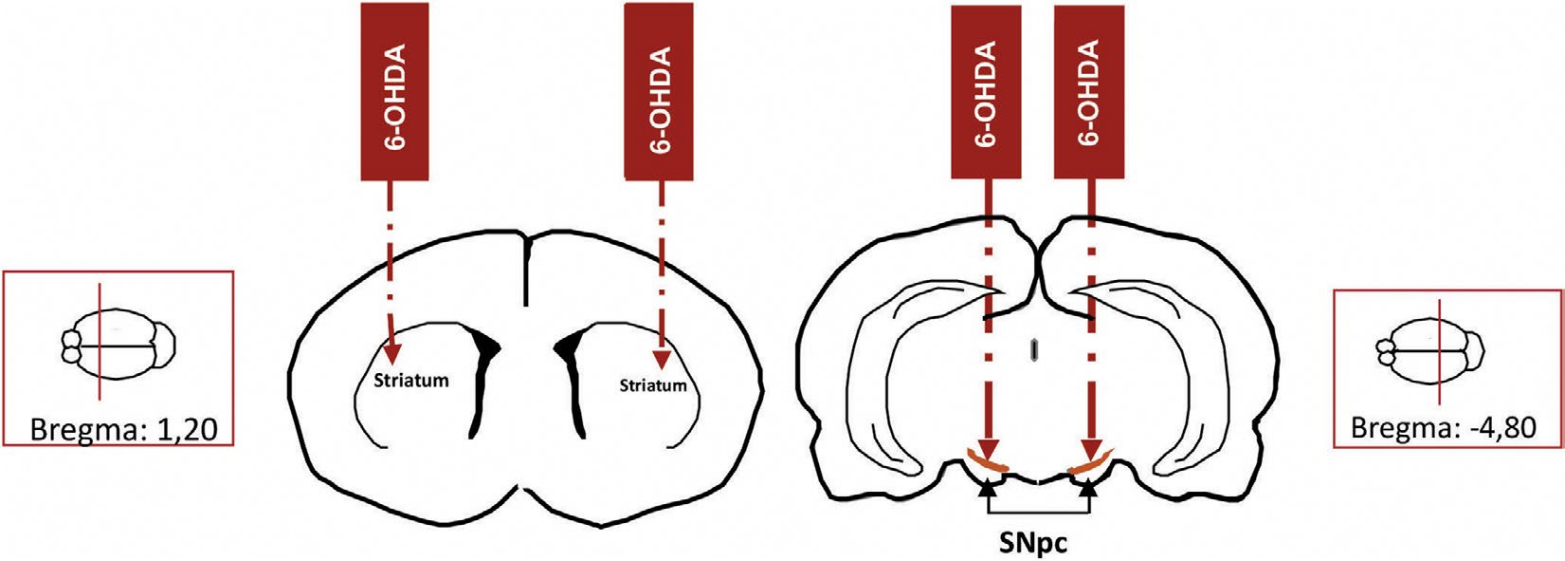
Diagrams of brain sites of 6‐OHDA infusions in the substantia nigra pars compacta (SNpc) and striatum based on coordinates from the Paxinos and Watson (2007) rat brain atlas.

Thirty minutes before stereotaxic surgery, the animals received an intraperitoneal (i.p.) injection of desipramine (25 mg/kg) to protect noradrenergic neurons (Padovan‐Neto et al. 2009). The animals were then anesthetized with Equitesin (3 mL/kg, i.p.; 1% sodium thiopental, 4.25% chloral hydrate, 2.13% magnesium sulfate, 42.8% propylene glycol, and 3.7% ethanol in water; Vieira et al. 2019), and the skulls were fixed in a stereotaxic frame (Kopf 957, Kopf Instruments, Tujunga, CA, USA). Bilateral infusions of 6‐OHDA in a total volume of 1 μL were made in the SNpc or dorsolateral striatum using a 27‐gauge stainless‐steel needle that was connected to a 5 μL Hamilton syringe (Hamilton Company, Reno, NV, USA) according to the following coordinates (Paxinos and Watson 2007): SNpc (anterior/posterior, −5.0 mm from bregma; medial/lateral, ±2.1 mm from midline; dorsal/ventral, −8.0 mm from the skull), striatum (anterior/posterior, +0.2 mm from bregma; medial/lateral, ±3.5 mm from midline; dorsal/ventral, −4.8 mm; Figure 1). The flow of the infusions was controlled by an electronic pump (Insight, Ribeirão Preto, Brazil) at a rate of 0.25 μL/min over 4 min (Bonato et al. 2018). The injection needle was left in place for an additional 2 min to avoid reflux. Sham surgery followed the same procedure, but the vehicle (sterile saline) was injected instead of 6‐OHDA. After surgery, the surgical wound was cleaned with chlorhexidine (Sigma‐Aldrich, St Louis, MO, USA), and the animals were individually placed in clean cages on warming pads until recovery. No analgesic or anesthetic antagonists were administered because these treatments could interfere with lesion development and thus functional outcomes. After recovery, the rats were returned to group housing (Padovan‐Neto et al. 2009). For 7 days following surgery, the animals had free access to laboratory rat chow that was moistened with water to make a paste. The rat chow and water were placed inside the cages to make them easily available and allow the animals to recover.

### 2.4 Experimental Design

**FIGURE 2:**
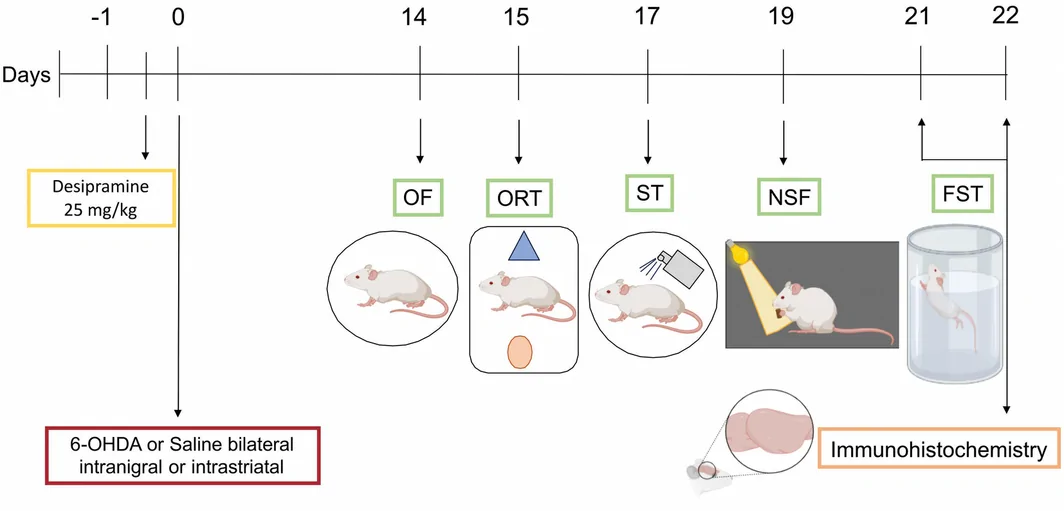
Experimental design. Thirty minutes before stereotaxic surgery and 6‐hydroxydopamine (6‐OHDA) infusions, the rats received 25 mg/kg desipramine intraperitoneally to protect noradrenergic terminals. 6‐OHDA was bilaterally injected in the substantia nigra pars compacta (SNpc) or striatum according to coordinates from the Paxinos and Watson (2007) rat brain atlas. Behavioral testing was conducted from day 14 to day 22 after the 6‐OHDA injections. The animals were euthanized on day 22, and their brains were removed for histological and biochemical analyses. FST, forced swim test; NSF, novelty‐suppressed feeding test; OF, open field test; ORT, object recognition test; ST, splash test.

The animals were randomly distributed into four experimental groups: SNpc saline (*n* = 12), SNpc 6‐OHDA (*n* = 39), striatum saline (*n* = 12), and striatum 6‐OHDA (*n* = 18). Behavioral testing was conducted from 8:00 AM to 12:00 AM. The OFT, ORT, ST, NSF, and FST were performed on days 14, 15, 17, 19, and 22 after the 6‐OHDA injections, respectively (Figure 2). All of the animals' behaviors were monitored and recorded using a video camera that was positioned above the apparatuses. The images were later analyzed using the ANYmaze contrast‐sensitive video monitoring system (Stoelting, Wood Dale, IL, USA). After behavioral testing, the animals' brains were removed and processed for histological analyses.

### 2.5 Behavioral Testing

#### 2.5.1 Open Field Test

The open field test evaluates locomotion and exploratory behavior (Gellért and Varga 2016). The OF consisted of a circular arena (80 cm diameter, 40 cm high wall). On day 14 after the lesion, each rat was gently moved from its home cage and immediately placed in the center of the OF. The rat was allowed to freely explore the apparatus for 5 min. The distance traveled (in meters), number of entries, and time spent in the center of the arena were recorded.

#### 2.5.2 Object Recognition Test

The object recognition test is based on the rodent's instinctive preference for novelty, thus allowing assessments of an animal's recognition memory (Akkerman et al. 2012). On the first day, the animals were placed on a round platform (80 cm diameter, 40 cm high wall) for 5 min for habituation. After two identical objects were placed symmetrically on the platform, the animal was given another 5 min for free exploration. Twenty‐four hours later, the animals were returned to the platform with the objects from the previous day, and they were allowed to explore them for 4 min. After a 1 h interval, the animals were returned to the platform, but one of the objects was replaced with a new object, which started the test session. Each animal was given 4 min to explore the new and old (familiar) objects. The exploration time for each object was recorded. Exploration was considered when the animal was at least 2 cm from the object or touched it. Animals that had an exploration time of fewer than 5 s were discarded from the test because this memory assessment requires sufficient exploratory behavior for reliable performance (Akkerman et al. 2012). The following parameters were evaluated: time spent by each animal exploring the familiar object and time spent exploring the new object. The exploration index (D2) was calculated using the following formula: (new object exploration time—familiar object exploration time) / (new object exploration time + familiar object exploration time).

#### 2.5.3 Splash Test

The splash test is used to evaluate motivational deficits and self‐care difficulties in rodents (Marques et al. 2019). In this test, grooming behavior, which can be considered an indirect measure of a hedonic state, was assessed. Each rat was individually placed on a circular platform (39 cm diameter). After 3 min of free exploration, a 10% sucrose solution was squirted on the dorsal coat of the rats. The latency to the first grooming and time spent grooming were recorded for 5 min.

#### 2.5.4 Novelty‐Suppressed Feeding Test

The novelty‐suppressed feeding has been used to assess anxiety‐like behaviors (Blasco‐Serra et al. 2017). This test introduces an additional element of motivation, in which the animal is deprived of food for a long period. Twenty‐four hours before the test, the animal's food was removed from the cage. The next day, the test was performed on a square platform (70 cm × 70 cm) on which each animal was placed individually. The room lights were turned off, and a spotlight was directed only toward the center of the platform on a food pellet. The animal was allowed to remain on the platform for 10 min or until the moment when it picked up the pellet. The animal was then transferred to a home cage with previously weighed food pellets and allowed to feed for 10 min. After this period, the food was removed and weighed again to record consumption. After the end of the test, the animals were returned to their cages with food and water available *ad libitum*. The following parameters were analyzed: latency (time for the animal to pick up the food) and consumption in the home cage (weight of the food before feeding—the weight of the food after feeding). The parameters were quantified manually.

#### 2.5.5 Forced Swim Test

The forced swim test identifies passive coping strategies in rodents (Can et al. 2011). The test was conducted in two sessions. In the training session, the rats were placed in a tank (25 cm diameter, 65 cm height) that was filled with water at a temperature of 24°C ± 1°C and depth of 30 cm for 15 min. Twenty‐four hours after the training session, the rats were subjected to the test session for 5 min, which was videotaped for subsequent quantification of the latency and immobility time (in seconds; that is, the absence of motion of the entire body and only small movements necessary to keep the animal's head above the water). The water was changed after each animal to avoid the influence of urinary or fecal material.

### 2.6 Immunohistochemistry

Five to eight animals were randomly chosen from each experimental group, and their brains were processed for immunohistochemistry. Briefly, the animals were deeply anesthetized with sodium thiopental (50 mg/kg; Thiopentax, Cristália, SP, Brazil) and then transcardially perfused with phosphate‐buffered saline (PBS) followed by 4% paraformaldehyde in 0.2 M phosphate buffer. The brains were removed, postfixed in the same fixative for 2 h, and cryoprotected by immersion in 30% sucrose for 48 h before snap freezing. Frozen tissue was serially sectioned on a cryostat (Cryocut 1800, Reichert‐Jung, Heidelberg, Germany) into 30 μm coronal sections that were collected in replicates in Eppendorf tubes that contained 0.01 M PBS with 0.01% sodium azide.

The brain sections were quenched in 1% H_2_O_2_ for 30 min and then blocked with 2% bovine serum albumin (BSA) in PBS for 60 min. The sections were incubated with mouse polyclonal anti‐TH antibody (1:2000, catalog no. AB152, Merck Millipore, Darmstadt, Germany) or goat polyclonal anti‐DCX antibody (1:1000, catalog no. sc‐8066, Santa Cruz Biotechnology, Santa Cruz, CA, USA) in PBS that contained 0.3% Triton X‐100 for 48 h at 4°C. The sections were then incubated with respective biotinylated secondary antibodies (1:500, anti‐mouse [catalog no. sc2039] and anti‐goat [catalog no. sc‐2042], Santa Cruz Biotechnology, Santa Cruz, CA, USA) for 2 h. The signals were visualized using avidin‐biotin complex (Vector Laboratories, Burlingame, CA, USA), 3,3′‐diaminobenzidine (DAB) as the chromogen, and 0.05% H_2_O_2_. The sections were then mounted on gelatin‐coated slides, dehydrated in ethanol, and coverslipped with Permount mounting medium.

For analysis, the slides were previously coded, and the analyses were performed by an experimenter who was blind to treatments. The slides were analyzed with an optical microscope (Olympus, BX41, PA, USA) that was attached to an Olympus QColor camera. For the quantification of TH‐IR cells, photomicrographs were obtained from the SNpc, VTA, and striatum with a 20× objective. ImageJ software (National Institutes of Health, Bethesda, MD, USA) was used to count the number of TH‐IR cells. The number of TH‐IR cells was obtained over the entire extension of the SNpc and VTA. For the striatum, measurements of integrated optical density (IOD) were performed in prefixed areas (0.58 mm^2^) that were previously defined. After acquiring the images, they were converted to a 16‐bit grayscale image, the background was removed, the threshold for the positive signal was defined, and the IOD was calculated. The two cerebral hemispheres were evaluated, and the average between them was obtained. The analysis was conducted with four to five sections per animal.

Doublecortin‐IR cells were counted throughout the SGZ of the dentate gyrus of the hippocampus (bregma −3.14 to −4.52 mm; Paxinos and Watson 2007). Five to six sections were analyzed per animal. For each section, the DG area was obtained, and the number of DCX‐IR cells was counted. Doublecortin‐IR neurons were classified into six different categories according to their dendrite morphology (Plümpe et al. 2006): A (neuron with no processes), B (neuron with very short processes), C and D (neurons with intermediate processes, with D being more developed than C), and E and F (which corresponded to more mature neurons; E neurons had apparent branches in the molecular layer of the SGZ, and F neurons had several branches along the dendritic tree). The results are presented as the sum of A + B (proliferative stage), C + D (intermediate stage), and E + F (post‐mitotic stage; Figure 5E).

### 2.7 Statistical Analysis

Statistica 8.0 software (StatSoft, Palo Alto, CA, USA) was used for the statistical analysis. A mortality curve was constructed to analyze survival data, and the log‐rank test (*χ* ^*2*^ test) was used to assess differences in animal survival. The behavioral and histological data were examined to verify normality (Shapiro–Wilk test) and homoscedasticity (*F* test to compare variances). Subsequently, the data were analyzed using Student's *t*‐test. The data are expressed as mean ± SEM. Values of *p* < 0.05 were considered statistically significant. Animals exhibiting outlier data points, as determined by predefined statistical criteria, were excluded from the final analysis using the software *Extreme values*, to ensure the robustness and reliability of the results.

## 3 Results

### 3.1 Bilateral 6‐OHDA Infusions in the SNpc or Striatum Cause Neurodegeneration of Dopaminergic Neurons in the SNpc, Striatum, and VTA

**FIGURE 3:**
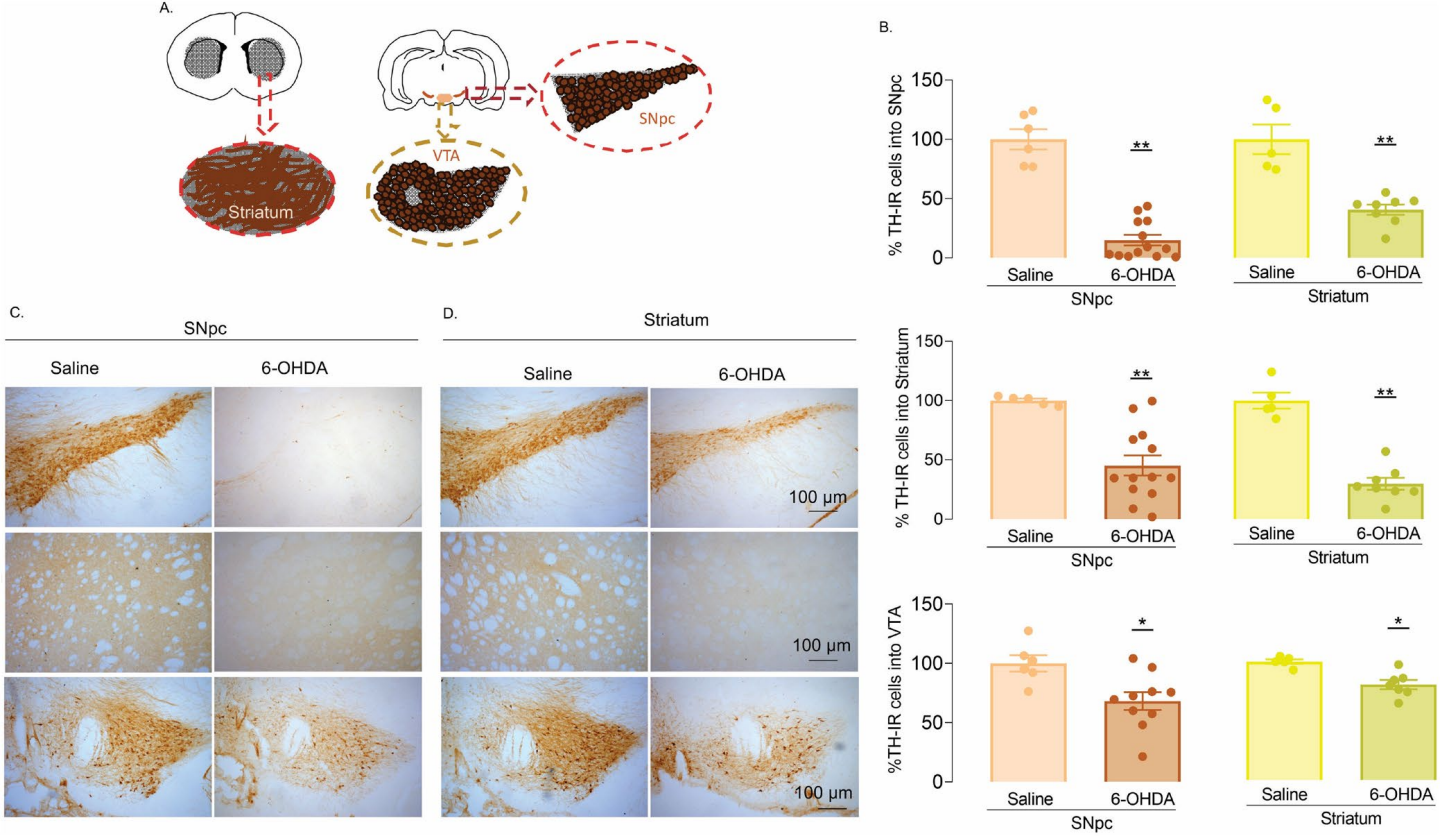
Bilateral infusions of 6‐OHDA in the SNpc and striatum result in the neurodegeneration of dopaminergic neurons in the SNpc, striatum, and VTA. (A) Representative diagrams of TH‐IR cells in the SNpc, striatum, and VTA. (B) Number of TH‐IR cells in the SNpc, striatum, and tegmental ventral area (TVA), represented as a percentage of controls. (C, D) Representative photomicrographs of TH‐IR cells in the SNpc (C) and striatum (D) 22 days after the 6‐OHDA injection. The data are expressed as a percentage of the control groups.

The degeneration of dopaminergic neurons was detected 22 days after the 6‐OHDA infusions (Figure 3). Six animals were randomly chosen to represent the control‐sham group because this group did not exhibit dopaminergic lesions. All animals that were infused with 6‐OHDA either in the SNpc or striatum were evaluated for TH immunoreactivity. For rats that received 6‐OHDA in the SNpc, six of 19 (31%) rats did not exhibit bilateral lesions. For rats that received bilateral infusions of 6‐OHDA in the striatum, seven of 15 (46%) rats did not exhibit bilateral lesions. Rats that did not exhibit bilateral lesions were removed from the histological and behavioral analyses. Compared with saline, bilateral 6‐OHDA injections in the SNpc decreased TH immunoreactivity in the SNpc (*t* = 9.8, *p* < 0.0001), striatum (*t* = 3.93, *p* < 0.001), and VTA (*t* = 2.86, *p* = 0.01). Similar effects were observed after 6‐OHDA injections in the striatum (*t* = 3.95–8.25, *p* < 0.01).

### 3.2 Bilateral 6‐OHDA Infusion Into the Striatum but Not in the SNpc Decreases the Locomotor Activity of Rats

**FIGURE 4:**
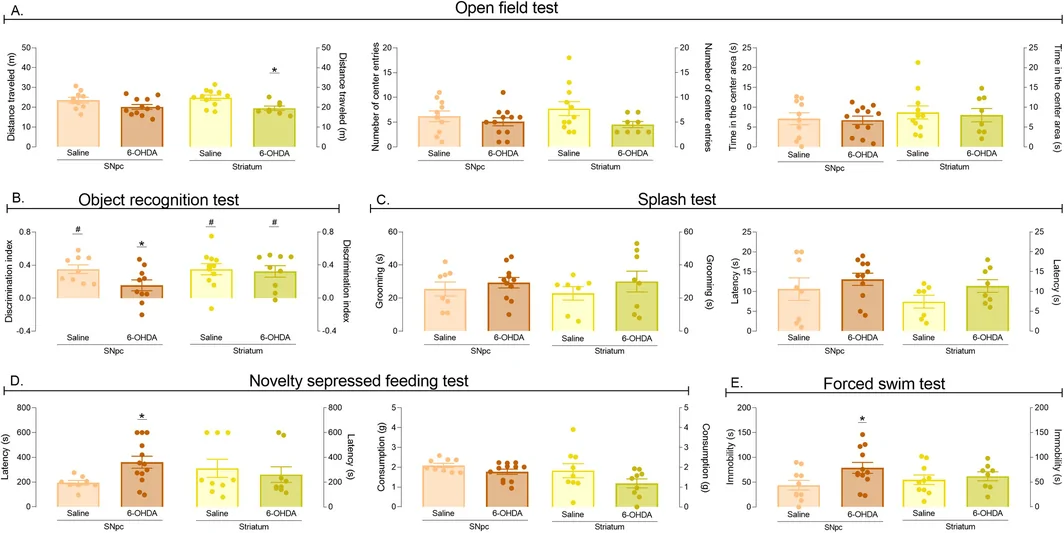
Behavioral effects of 6‐OHDA infusions in the SNpc and striatum in rats. (A) Distance traveled (in meters), time in the center area (in seconds), and number of center entries in the OFT 14 days after the 6‐OHDA injection. (B) Bilateral intranigral infusions of 6‐OHDA affected recognition memory in rats in the ORT. Recognition memory was evaluated with a 1 h interval between the training session and test session. (C) No effects of 6‐OHDA infusions in the SNpc or striatum were observed in rats in the splash test. Bilateral intranigral 6‐OHDA injections increased the latency to feed in rats in the NSF. (D) Latency to feed (in seconds) and consumption in the home cage were recorded for 10 min. (E) Bilateral intranigral infusions of 6‐OHDA increased immobility time in rats in the FST. Immobility was recorded for 5 min in the FST. The data are expressed as the mean ± SEM in the saline SNpc group (*n* = 10), 6‐OHDA SNpc group (*n* = 12), saline striatum group (*n* = 11), and 6‐OHDA striatum group (*n* = 9). *p < 0.05, compared with saline striatum group (*t*‐test); ^#^ *p* < 0.05, compared with zero in the ORT.

As seen in Figure 4A direct infusion of 6‐OHDA into the striatum resulted in a significant reduction of locomotor activity of lesioned animals when compared to controls (*t* = 3.02, *p* = 0.007). No significant difference was detected between animals that received 6‐OHDA infusion into the SNpc when compared to those that received saline into the same brain region (*t* = 1.87, *p* = 0.08). The other OF parameters, that is, the number of entries and time in the center were not affected by 6‐OHDA infusion into the SN or into the striatum when compared with control groups (*p* > 0.05; Figure 4A).

### 3.3 Bilateral 6‐OHDA Infusion Into the SNpc but Not in the Striatum Affects the Recognition Memory in the ORT

Fifteen days after surgery animals were evaluated in the ORT (Figure 4B). To investigate which groups had functional recognition memory, the D2 value in the ORT was compared to zero. Groups that received saline into the SNpc or striatum, discriminated against the different objects very well (*t* = 5.18–5.41, *p* < 0.0001). This ability for functional recognition memory was impaired in the group that received 6‐OHDA into the SNpc (*t* = 1.95, *p* > 0.05, compared to zero), but not into the striatum (*t* = 6.25, *p* < 0.0001), compared to zero. When compared with the control treatment (saline), 6‐OHDA reduced the exploration index when injected into the SNpc (*t* = 2.27, *p* < 0.05; Figure 4B), but not into the striatum (*t* = 0.27, *p* > 0.05), further indicating the differential effect of 6‐OHDA on object recognition memory as it is injected in the SNpc or striatum.

### 3.4 Bilateral 6‐OHDA Infusion in the SNpc or Striatum Evaluated in the Splash Test

Figure 4C shows the effects of bilateral intranigral or intrastriatal infusion of 6‐OHDA on the Splash test. There was no significant difference between groups in the grooming or latency when the 6‐OHDA was injected into SNpc (*t* = 0.42–0.73, *p* > 0.05) or striatum (*t* = 0.92–1.73, *p* > 0.05).

### 3.5 Bilateral 6‐OHDA Intranigral Infusion Increases the Latency to Feed in the NSF

Figure 4D shows the increase in the latency to feed of animals that received 6‐OHDA infusion in the SNpc when compared with the saline group 19 days after stereotaxic surgeries (*t* = 2.60, *p* = 0.02). A similar effect did not occur, however, when 6‐OHDA was injected into the striatum (*t* = 0.52, *p* > 0.05, vs. saline). There was no significant effect in the consumption parameter when compared to the animals that received 6‐OHDA into the SNpc or the striatum with the saline group (*t* = 1.52–1.80, *p* > 0.05; Figure 4D).

### 3.6 Bilateral 6‐OHDA Intranigral Infusion Increases the Immobility Time in the FST

Lesioned animals with 6‐OHDA in the SNpc showed an increase in immobility time when compared with the saline group (*t* = 2.20, *p* < 0.05; Figure 4E). No change occurred in immobility time, however, when 6‐OHDA was injected into the striatum (*t* = 0.51, *p* > 0.05).

### 3.7 Bilateral Intrastriatal 6‐OHDA Infusion but Not in the SNpc Reduces Hippocampal DCX Cell Population and Impacts the Postmitotic Stage of Newborn Neurons

**FIGURE 5:**
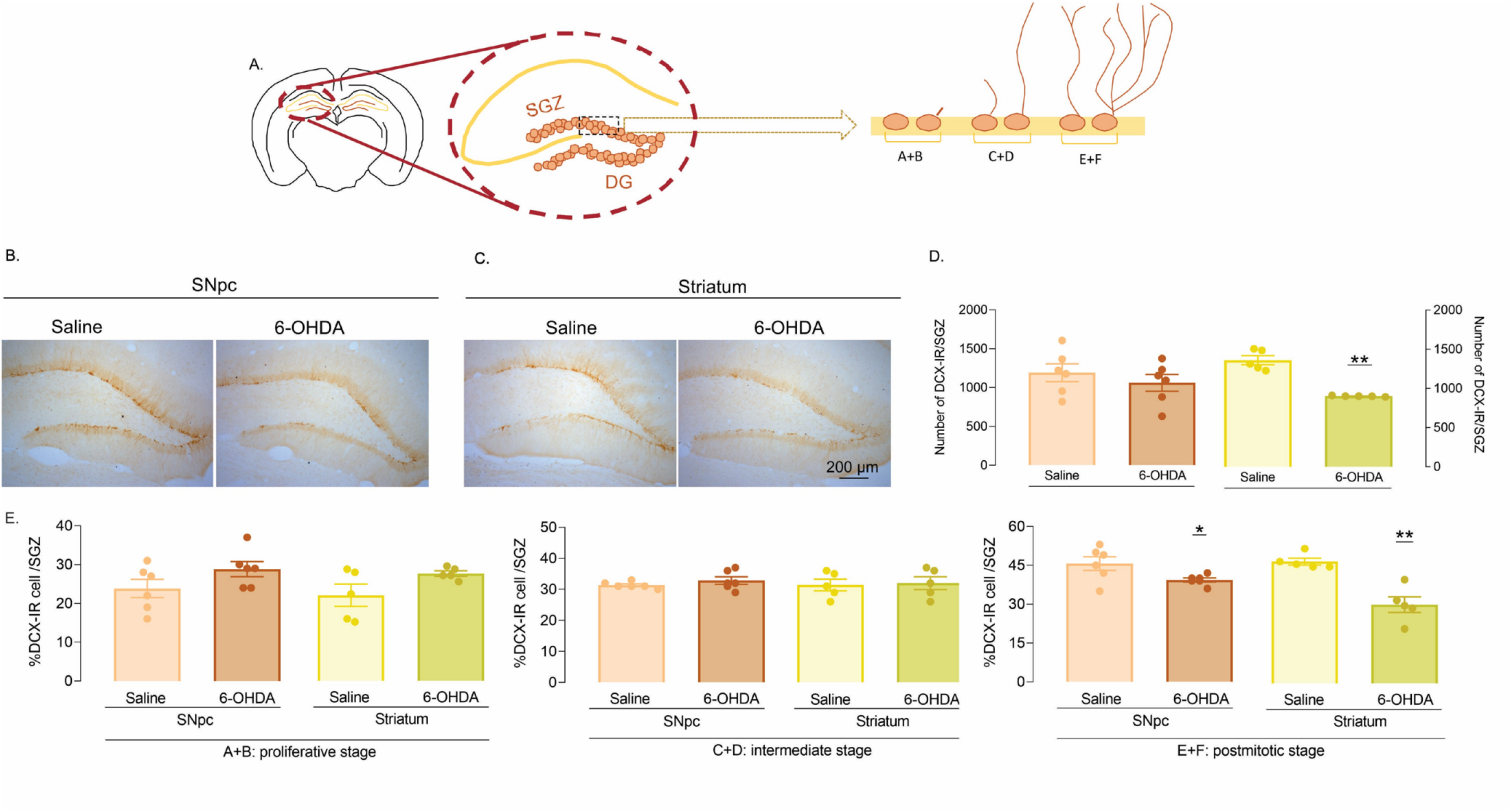
Bilateral 6‐OHDA injections in the striatum reduce hippocampal DCX cell population, and impact neuronal maturation. (A) Representative diagram of DCX‐IR cells into the SGZ of the dentate gyrus of the hippocampus and classification of dendritic morphology. (B, C) Representative photomicrographs of DCX‐IR cells in the hippocampus after 6‐OHDA infusions in the SNpc (B) and striatum (C). (D) The number of DCX‐IR cells in the SGZ of the dentate gyrus of the hippocampus. (F–H) Classification of DCX‐IR cells in the SGZ according to the stage of neuronal maturation (proliferative, intermediate, and postmitotic). The data are expressed as the mean ± SEM in the saline SNpc group (*n* = 6), 6‐OHDA SNpc group (*n* = 6), saline striatum group (*n* = 5), and 6‐OHDA striatum group (*n* = 5). **p < 0.001, compared with controls (*t*‐test).

Figure 5 shows the effects of 6‐OHDA infusion into the striatum or SNpc on hippocampal DCX cell population. Compared with saline, 6‐OHDA reduced the number of DCX‐IR cells in the SGZ of hippocampal DG when it was injected into the striatum (*t* = 8.02, *p* < 0.0001) but not into the SNpc (*t* = 0.82, df = 10, *p* = 0.43, Figure 5D). Compared to saline, 6‐OHDA reduced the percentage of DCX‐IR cells in the postmitotic stage when injected either into the SNpc (*t* = 2.28, df = 10, *p* = 0.04) or striatum (*t* = 5.01, df = 8, *p* = 0.001) (Figure 5E). There was no effect of 6‐OHDA on the proliferative and intermediate stages of DCX cells development, whether it was injected into the SNpc or striatum (*t* = 0.21–1.89, *p* > 0.05).

## 4 Discussion

We investigated cognitive and affective symptoms following 6‐OHDA infusions in the SNpc and striatum in rats. Our objective was to determine the most appropriate brain site for 6‐OHDA infusions for future investigations of neuroprotective pharmacotherapies for nonmotor symptoms of PD. 6‐OHDA infusions in either the SNpc or striatum caused the massive degeneration of TH‐IR neurons in the SNpc and striatum and less extensively in the VTA. About functional outcomes, 6‐OHDA infusions in the striatum resulted in a decrease in general exploratory activity (i.e., distance traveled in the OFT) 14 days after the lesion. Rats that received 6‐OHDA in the SNpc exhibited cognitive impairments in the ORT and despair‐like behavior in the FST. No other significant behavioral changes were detected after 6‐OHDA infusions in the SNpc or striatum over the 22 days. A decrease in the number of DCX‐IR neurons was found in the hippocampus in rats that received 6‐OHDA in the striatum, indicating a decrease in reduced number of immature neurons. 6‐OHDA infusions in both the SNpc and striatum impacted the maturation of newborn hippocampal neurons. This effect might weaken the establishment of synaptic connectivity and integration of these neurons in hippocampal circuitry, impacting hippocampus‐mediated functions.

The mechanism of 6‐OHDA lesions involves mitochondrial dysfunction and an increase in oxidative stress that, in turn, leads to neuronal degeneration. In the present study, we found that bilateral intranigral 6‐OHDA infusions in the SNpc or striatum resulted in 69.2% and 33.3% mortality over time, respectively. The degree of dopaminergic neurodegeneration after 6‐OHDA infusions in the SNpc was more marked (85%) than 6‐OHDA infusions in the striatum (50%). These results are consistent with previous findings that 6‐OHDA infusions in the SNpc resulted in massive neurodegeneration of the nigrostriatal pathway and high mortality rates (Simola et al. 2007; Blandini and Armentero 2012; Bonato et al. 2018).

In the present study, 6‐OHDA‐lesioned animals exhibited different functional outcomes depending on the site of the toxin injection. 6‐OHDA infusions in the SNpc resulted in a decrease in the discrimination index in the ORT, an increase in latency in the NSF, and an increase in immobility in the FST, indicating cognitive deficits and emotional impairments that were induced by the toxin infusion. No motor effect was detected in rats that received 6‐OHDA in the SNpc 14 days after the lesion. Otherwise, rats that received 6‐OHDA in the striatum exhibited a decrease in general locomotor activity in the OFT. The reason for this discrepancy between motor activity of rats is unkown. Experimental evidence has indicated that a complete (> 95%) destruction of nigrostriatal dopamine is necessary to produce permanent motor, sensory, and cognitive deficits from which animals do not recover (Robinson et al. 1994). Only 10%–20% of the dopamine input to the striatum is required to maintain relatively normal function. Although animals with extensive but partial depletion (80%–95%) of striatal dopamine initially exhibit severe behavioral deficits, most of these animals regain many of their behavioral capacities and recover their function over time (Marshall 1979; Robinson et al. 1994). Therefore, rats with bilateral 6‐OHDA lesions may have recovered from this injury 14 days after the lesion. Supporting this possibility, intra‐SNpc 6‐OHDA infusions decreased general locomotion in rats 24 h after the lesion, but no effect was detected 14 days after the 6‐OHDA infusion in the SNpc (Bonato et al. 2018). Additionally, intranigral 6‐OHDA infusions might cause a lack of habituation to novelty in the OF. Although no significant alterations in spontaneous locomotor activity were observed in the open field test, subtle motor coordination impairments cannot be excluded, since this paradigm is not a specific assessment of fine motor function. This effect has been related to changes in hippocampal function, in which the hippocampus is important for the recognition of novel environments (Leussis and Bolivar 2006). The lack of habituation to novelty was also described in a Parkin‐deficient mouse model of PD (Rial et al. 2014).

Bilateral infusions of 6‐OHDA in the SNPc have been shown to produce dopaminergic neurodegeneration and consistent depressive‐like behaviors in rats (Santiago et al. 2010; Bonato et al. 2018). In the present study, 6‐OHDA‐lesioned rats exhibited memory deficits in the ORT compared with controls.

The novelty‐suppressed feeding test has been used as a measure of anxiety‐ and depressive‐like behaviors (Blasco‐Serra et al. 2017). Intranigral infusions of 6‐OHDA promoted an increase in the latency to feed in the NSF 18 days after the lesion. This finding was accompanied by another feature of behavioral despair, reflected by an increase in immobility time in the forced swim test. Cannabidiol attenuated effects of 6‐OHDA lesions in both the NSF test and FST, indicating an antidepressant‐like effect of this treatment. Indeed, CBD has been shown to promote antidepressant‐like effect in several experimental conditions. Although the pharmacological mechanism are not entirely clear, the antidepressant‐like effect of CBD has been related to serotonin 5‐hydroxytryptamine‐1A (5‐HT_1A_) receptor activation (Zanelati et al. 2010; Sales et al. 2018).

The degeneration of midbrain dopaminergic neurons has been shown to affect several mesolimbic structures, including the hippocampus (Bonito‐Oliva et al. 2014; Weerasinghe‐Mudiyanselage et al. 2022), which is critically involved in memory and emotional control. Hippocampal dysfunction has been reported in neurotoxic and genetic animal models of PD, including impaired long‐term potentiation (LTP) induction and maintenance in the CA1 hippocampal region, reduced PSD‐95 expression, and altered dopamine D1 receptor expression in 6‐OHDA‐lesioned rats (Esmaeili‐Mahani et al. 2021), as well as in *α*‐synuclein transgenic mice (Costa et al. 2012). Since LTP induction in the hippocampus plays a key role in memory formation (Nabavi et al. 2014; McNaughton and Morris 1987), hippocampal LTP deficits could also be involved in 6‐OHDA‐induced learning and memory impairments. The loss of dopaminergic neurons induced by intranigral 6‐OHDA infusions was accompanied by an area‐dependent degeneration of noradrenergic and serotonergic systems, characterized by alterations in hippocampal norepinephrine and serotonin levels, and a decline of hippocampal synaptic plasticity, which were associated with depressive‐like and anxiety‐like behavior (Santiago et al. 2010; Delaville et al. 2012; Marxreiter et al. 2013; Lim et al. 2018).

Hippocampal neurogenesis is a common feature between rodents and humans and has been considered particularly important for hippocampus‐dependent functions, such as cognition and emotion (Anacker and Hen 2017). A reduction of hippocampal neurogenesis has been linked to the expression of anxiety‐ and depressive‐like symptoms. During adult neurogenesis, newly generated neurons pass through a stage that is characterized by the expression of DCX, a microtubule‐associated protein, beginning in the initial stages of neuronal differentiation and persisting until more advanced stages, including dendrite maturation and branching (Brown et al. 2003). A decrease in the total number of DCX‐IR neurons was detected in the SGZ after 6‐OHDA infusions in the striatum. Unexpectedly, no significant change was observed in the number of DCX‐IR neurons in the SGZ after 6‐OHDA was injected into the SNpc. Dopaminergic fibers that originate from the VTA and SNpc directly innervate the hippocampus and cortex, suggesting functional and anatomical links between those dopaminergic pathways and hippocampus‐dependent functions (Barzilai and Melamed 2003; Alexander 2004).

Although dopaminergic signaling is known to modulate hippocampal plasticity and adult neurogenesis, the effects of dopamine depletion on DCX cells are likely mediated by broader circuit‐level and neurotransmitter network changes rather than by a direct and linear relationship with the site of dopaminergic lesion (Song et al. 2012; Mu et al. 2011). A possible explanation for the stronger reduction of DCX cells in the intrastriatal model is that this lesion primarily disrupts cortico–basal ganglia–thalamo–cortical circuits, which may alter hippocampal activity through cortical inputs, particularly from the prefrontal and entorhinal cortices via the perforant pathway (Song et al. 2012). Since hippocampal neurogenesis is activity‐dependent, such network dysfunction may negatively impact the survival and maturation of DCX neuroblasts. In addition, intrastriatal 6‐OHDA induces progressive retrograde degeneration of nigrostriatal neurons, which may result in prolonged dysregulation of neurotransmitter systems involved in the control of adult hippocampal neurogenesis, including serotonergic and noradrenergic pathways. Furthermore, dopaminergic lesioning has been associated with the accumulation of truncated and insoluble *α*‐synuclein in the hippocampus, accompanied by increased calpain‐1 activity, suggesting additional pathological mechanisms that may impair neuroblast survival and maturation (Schlachetzki et al. 2016). Finally, the reduction in DCX^+^ cells likely reflects alterations in the maturation and survival of immature neurons rather than in early progenitor proliferation, since dopamine appears to play an important role during later stages of neuronal maturation and synaptic integration (Mu et al. 2011).

When we analyzed the distribution of DCX‐IR cells at different stages of dendrite development/maturation, we found that 6‐OHDA lesions preferentially decreased DCX‐IR neurons in the postmitotic stage. This result suggests that 6‐OHDA infusions in the SNpc or striatum impact neuronal maturation in the hippocampus, which would weaken the establishment of synaptic connectivity and integration of these neurons in hippocampal circuitry. These effects might impact cognitive and emotional functions in 6‐OHDA‐lesioned animals.

In conclusion, the bilateral injection of 6‐OHDA into the SNpc resulted in more pronounced behavioral (cognitive and emotional) alterations compared with the intrastriatal injection. In addition, the SNpc model promoted a more severe dopaminergic neurodegeneration than the intrastriatal model, although both models were associated with alterations in neuronal maturation in the hippocampus.

### 4.1 Study Limitation

We acknowledge that this study has certain limitations that should be addressed in future research to further advance the understanding of PD pathology. First, only male rats were evaluated, which precluded the analysis of potential sex‐related influences on behavioral and neurogenic outcomes. Additionally, although bilateral 6‐OHDA lesions are a well‐established model for studying PD, they do not fully capture the progressive and multifactorial nature of the human condition and do not produce α‐synuclein aggregates, a hallmark of the disease. Nevertheless, the study provides valuable insights into the differential behavioral and neurogenic consequences of bilateral 6‐OHDA lesions in the substantia nigra and striatum, supporting their use in investigating non‐motor symptoms of PD.

The findings of this study have potential translational implications for understanding non‐motor symptoms of PD and for developing targeted therapeutic strategies. By demonstrating differential behavioral and neurogenic effects of bilateral 6‐OHDA lesions in the substantia nigra and striatum, our results indicate that one of these lesion models may be particularly suitable for studying non‐motor symptoms of PD. Future research should explore these effects in female animals to assess sex‐related differences, extend the follow‐up period to evaluate long‐term outcomes, and consider models that incorporate *α*‐synuclein pathology to more closely mimic the progressive and multifactorial nature of human PD. Additionally, investigating pharmacological or non‐pharmacological interventions within these lesion models could help bridge preclinical findings to potential clinical applications. Looking to the future, it is suggested that other brain regions related to non‐motor outcomes be investigated, especially anxiety and depressive‐type behaviors, as well as further analyses aimed at understanding the neurobiological mechanisms involved in the observed changes, with an emphasis on neurogenesis processes.

## Author Contributions

Bianca Andretto de Mattos, Jessica Mendes Bonato and Rubia Maria Weffort de Oliveira conceived and designed the experiments and wrote the manuscript. Bianca Andretto de Mattos and Jessica Mendes Bonato performed the experiments. Humberto Milani helped with data and statistical analysis. Elaine Del Bel made a critical revision of the manuscript before submission to the Journal. All authors discussed and commented on the manuscript.

## Funding

This work was supported by Conselho Nacional de Desenvolvimento Científico e Tecnológico (CNPq), Universidade Estadual de Maringá (UEM), Paraná, Brazil, and Coordenação de Aperfeiçoamento de Pessoal de Nível Superior (CAPES). The authors thank Marcos Alberto Trombelli for his technical support.

## Ethics Statement

This study was carried out at the State University of Maringá in *strict* accordance with the Brazilian College of Animal Experimentation (COBEA) recommendations. Animal experiments were approved by the local Ethics Committee on Animal Experimentation of the State University of Maringá (animal license number: CEUA no. 8156290119).

## Consent

All of the co‐authors approved the final version of the manuscript and agreed to submit it to Neurotoxicity Research.

## Conflicts of Interest

The authors declare no conflicts of interest.

## Data Availability

The data that support the findings of this study are available from the corresponding author upon reasonable request.
